# Comparative Evaluation of the Ileum Microbiota Composition in Piglets at Different Growth Stages

**DOI:** 10.3389/fmicb.2021.765691

**Published:** 2021-12-01

**Authors:** Chang Lu, Yadan Liu, Yijia Ma, Shu Wang, Chunbo Cai, Yang Yang, Yan Zhao, Guoming Liang, Guoqing Cao, Bugao Li, Sung Woo Kim, Xiaohong Guo, Pengfei Gao

**Affiliations:** ^1^College of Animal Science, Shanxi Agricultural University, Taigu, China; ^2^Department of Animal Science, North Carolina State University, Raleigh, NC, United States

**Keywords:** Jinfen White and Mashen piglets, 16S rRNA, ileum microbiota, intestinal microorganism, growth stage

## Abstract

Intestinal microbiota can affect the intake, storage, and absorption of nutrients in the body, thereby greatly impacting the growth and development of animals. In addition to diet, the breed and growth stages of pigs could also affect changes in the intestinal microbiota. However, research on the developmental changes in the ileum microbiota of piglets remains unclear. In this study, the ileum microbiota of Jinfen White and Mashen piglets at different developmental stages were investigated using 16S rRNA sequencing. Physiologically, the villus height of the ileum decreased, and the crypt depth increased during the development of the two pig breeds. Additionally, the serum antioxidant factors in the Jinfen White piglets were significantly higher than in the Mashen piglets at the end of the nursing stage. A total of 690 operational taxonomic units (OTUs) belonging to 21 phyla and 286 genera were identified, of which Firmicutes and Proteobacteria were the dominant phyla during the development of both the Jinfen White and Mashen piglets, accounting for ∼90% of all OTUs. Further research revealed differences in dominant bacteria between the two breeds. With increasing age, the ileum microbial diversity increased, and in both the pig breeds, the proportion of Firmicutes increased, whereas the proportion of Proteobacteria decreased. Additionally, different samples were characterized by specific genera, and different Kyoto Encyclopedia of Genes and Genomes (KEGG) pathways were predicted at certain developmental stages. Finally, the correlation between the ileum microbiota and physiological features was analyzed, and it was suggested that the host and environmental factors play important roles in the formation of the microbial community structure in piglets. In summary, we delineated the structure, function, and differences in ileum microbiota between Jinfen White and Mashen piglets during different growth stages. This study helps to understand the development of the intestinal microbiota in local and hybrid pig breeds.

## Introduction

The intestinal tract is an important digestive and immune organ. Maintaining intestinal health is important for growth and disease prevention in pigs. To date, many studies have found that the intestinal microbiota are involved in the absorption and digestion of nutrients in their hosts (Judkins et al., 2020; Gill et al., 2021) and can consume, store, and redistribute energy to maintain the body’s dynamic balance during various growth stages (Hillman et al., 2017). The intestinal microbiota of pigs contains thousands of different microbial species, including Firmicutes, Bacteroidetes, and Proteobacteria (Holman et al., 2017). In recent years, high microbial diversity has been observed in the feces of piglets at birth, with the dominant phyla beings Bacteroidetes, Firmicutes, Metamorphetes, and Fusobacteria (Liu et al., 2019). The proportion of Firmicutes in the feces of older pigs (2-, 3-, and 6-months) was significantly higher than that from 1-month piglets, and the microbial composition tended to be more stable in the mature pigs (Zhao et al., 2015). To explore the association between pig breed and the fecal microbial community, Yang et al. (2021) investigated the differences in fecal microbial composition among different pig breeds. The intestinal microbiota of Landrace, Yorkshire, and Duroc pigs were highly similar, while those from Bama, Erhualian, and Xiaomeishan pigs from China were highly similar (Yang et al., 2014). Additionally, it was also found that the intestinal microbiota is easily affected by various factors, including age, diet, dietary fiber, antibiotics, and probiotics, and the dominant phyla are Firmicutes and Proteobacteria (Wang et al., 2019). Feed efficiency-related compositional differences in the intestinal microbiota were revealed by characterizing the intestinal microbiota of pig ranks as the different residual feed intake (RFI), suggesting that the intestinal microbiota is possibly linked to feed efficiency in pigs (McCormack et al., 2017). Dietary fiber is a beneficial nutrient for animal production and can prevent intestinal diseases. Chen indicated that the influences of dietary sources on intestinal microbiota and volatile fatty acids were related to fiber composition in finishing pigs. In addition, bran fiber and pea fiber can not only increase the *Lactobacillus* and *Bifidobacterium* populations in the intestine, but also increase the height of intestinal villi (Chen et al., 2014).

The development of high-throughput sequencing (HTS) technology has greatly promoted our understanding of intestinal microbial function, and research on the pig intestinal microbiota is gradually becoming a hot topic. Among them, 16S rRNA sequencing technology has been widely used in the study of the diversity and composition of animal intestinal microbiota, which provides a convenient technology for further research, including intestinal microbial research on Large White pigs, Chinese Shanxi Black pigs (Gao et al., 2019b), Duroc pigs (Si et al., 2020), and Meishan pigs (Cui et al., 2019). In addition, Mashen pigs are an excellent indigenous breed in Shanxi Province, which is characterized by a high fertility, good meat quality, strong resistance to stress, and a slow growth rate (Li et al., 2021). Jinfen White pigs are a local hybrid breed of multiple pigs, including Mashen (6.25%), Taihu (3.13%), Landrace (40.62%), and Yorkshire pigs (50%). They inherit the advantages of good quality meat and strong stress resistance from Mashen pigs, but their growth rate is significantly higher than that of Mashen pigs. In particular, after 70 days, the average daily weight gain in Jinfen White pigs was 863 g, while that of Mashen pigs was only 460 g, suggesting a significant difference in the nutrient absorption and metabolism in the intestines of these two breeds, and this difference may be more significant at the piglet stage. The intestinal system is not completely developed in piglets; moreover, the intestinal microbial diversity directly affects intestinal development and homeostasis (Frese et al., 2015). During development, the piglet stage is the key period of intestinal microbial colonization, which affects the nutritional intake, oxidative metabolism, and immune performance. Moreover, the intestinal structure is not fully developed and the intestinal microecology is in flux, and therefore, it is easily stressed by changes in the feeding environment (Guevarra et al., 2018, 2019). Several studies have been conducted on the intestinal microbiota of pigs, but the ileum microbiota composition and its role at different growth stages in Jinfen White and Mashen piglets remains unknown. Furthermore, the difference in the ileum microbiota between local and hybrid pig breeds has not been clarified to date.

Therefore, in this study, microbiota in the ileum segment of Jinfen White and Mashen piglets were sampled at three growth stages (18 samples): newborn (1 day), weaning (28 days), and end of nursing period (70 days). We investigated the composition of the ileum microbiota in Mashen and Jinfen White piglets using 16S rRNA sequencing and revealed possible correlations between the microbial community structure and different traits (serum biochemical indexes and villus features). This will advance our knowledge of the composition and predicted function of the ileum microbiota during the developmental stages and help link the intestinal microbiota with environmental traits.

## Materials and Methods

### Animal Management and Sample Collection

In this study, nine Jinfen White piglets and nine Mashen piglets were raised in the Datong Pig Breeding Farm (Shanxi Province, China). The piglets were weaned at the same age at 28 days and raised under the same nursery conditions. Both breeds of piglets were used for content sampling of the ileum at the newborn stage (1 day), weaning stage (28 days), and end of nursery stage (70 days). The feed formulation is provided in Supplementary Table 1. The piglets fasted for 12 h and were then allowed free access to fresh water prior to slaughter. The initial and final weight of each piglet are listed in Supplementary Table 2. The ileum samples were collected at three developmental stages, kept frozen in liquid nitrogen for transportation, and then stored at −80°C until use. The animal operating procedures involved in this study were approved by the Animal Care and Use Committee of Shanxi Agricultural University, and the methods were in accordance with the Guidelines for the Care and Use of Experimental Animals of the Ministry of Agriculture of China.

### Determination of Ileum Structure and Physiochemical Properties

The ileum was fixed with 4% paraformaldehyde for 48 h and then soaked in paraffin. The paraffin-soaked tissue was cut into 4 μm sections and deparaffinized for a further 1 h at 62°C. Then, the sample was stained with hematoxylin–eosin, observed, and photographed under an optical microscope. For each ileum sample, a paraffin-embedded section was prepared and Image-Pro Plus software (version 6.0) was used to measure the villus length, crypt depth, and the ratio of villus length to crypt depth (V/C). Three slices were selected from each pig, and three non-overlapping fields were randomly observed in each slice.

### Determination of Serum Biochemical Indexes of Piglets

Blood (5 mL) was collected from the vena cava before slaughter at different developmental stages of piglets, and serum was separated by centrifugation at 4000 r/min for 10 min at 4°C. Enzyme-linked immunosorbent assay (ELISA) was used to detect serum antioxidant factors, including superoxide dismutase (SOD), malondialdehyde (MDA), and total antioxidant capacity (T-AOC). All the above specific detection kits were purchased from Shanghai Enzyme-linked Biotechnology Co., Ltd.

### DNA Extraction and Sequencing

Microbial genomic DNA was extracted from the ileum of different individuals using the QIAamp Fast DNA Stool Mini Kit (Qiagen, Valencia, CA, United States) following the manufacturer’s instructions. The V3–V4 region of the 16S rRNA gene was amplified by polymerase chain reaction (PCR) using universal bacterial 16S rRNA gene PCR amplicon primers (341F: 5′-ACTCCTACGGGRSGCGCAG-3′, 806R: 5′-GGACTACWGGGTATCTAATC-3′), and the PCR products were purified using a DNA Gel Extraction Kit (Tiangen, Beijing, China). All sample libraries were sequenced on the Illumina HiSeq 2500 platform.

### Microbial Analysis

The 16S rRNA sequencing data were processed using the Quantitative Insights into Microbial Ecology (QIIME1) (version 1.9.1) platform (Caporaso et al., 2010). The sequence reads were acquired after trimming out the barcodes and primers and were discarded with a >10% ambiguous base (N) or 20% low-quality base pairs (*Q* < 20). Then, the raw reads were filtered with a minimum overlap of 10 bp and a maximum mismatch ratio of 0.2, using FLASH (version 1.2.11) (Magoc and Salzberg, 2011). The reads were clustered into operational taxonomic units (OTUs) using *de novo* picking, with a 97% sequence identity threshold, and chimeras and singletons were removed using USEARCH software (version 7^1^). Subsequently, OUTs were aligned to the SILVA rRNA specific database^2^ to assign taxonomy, and a phylogenetic tree was generated within QIIME. The microbial alpha diversity indices of the samples were determined using Chao1, ACE, and Sobs (which measures richness based on rare OTUs), coverage, Shannon, and Simpson (which measure richness and evenness), and these indices were calculated using the Mothur program.^3^ Principal coordinate analysis (PCoA) plots, based on unweighted UniFrac distances, were visualized using EMPeror v0.9.3-dev. Subsequent downstream images were generated using the R package Phyloseq. Tax4Fun v1.0, which employs the 16S rRNA gene as a maker by using the Silva databases for taxonomy and OUT assignments, was used to predict the functionality of the ileum microbiota of the two pig breeds (Asshauer et al., 2015). The prediction of functions was inferred based on the Kyoto Encyclopedia of Genes and Genomes (KEGG) annotations for level 2 pathways. Pathways for which the relative abundance was <0.001% were dismissed.

### Data Availability

The 16S rRNA gene sequence data used in this study were deposited in the NCBI SRA database under the accession number SRP321842.

### Statistical Analysis

The physiological features (villus height and crypt depth) and serum antioxidant factors are presented as mean ± SEM, and statistical analyses were performed using SPSS ver.26.0. Differences between the Jinfen White piglets and Mashen piglets were analyzed using Student’s *t*-test; ^∗∗^ and ^∗^ indicate that the differences between the samples are extremely significant (*P* < 0.01) or significant (*P* < 0.05), respectively. Differences at different time points were identified using ANOVA; values with the same or no letter superscripts mean no significant difference (*P* > 0.05), those with different small letter superscripts mean a significant difference with *P* < 0.05, and those with different capital letter superscripts mean a significant difference with *P* < 0.01.

## Results

### Physiological Features of Ileum in Piglets

**FIGURE 1:**
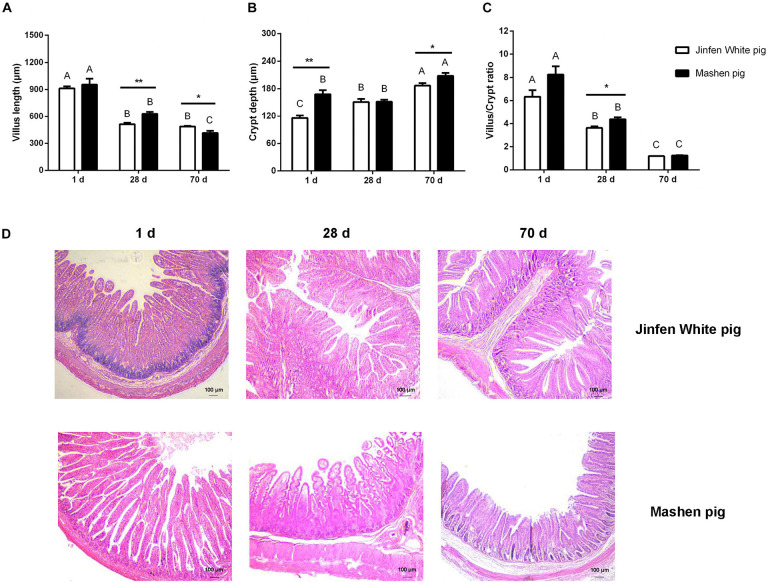
The intestinal morphology of Jinfen White and Mashen piglets. **(A)** The villus height (μm), **(B)** crypt depth (μm), **(C)** villus/crypt ratio, and **(D)** ileum intestinal biopsy of Jinfen White and Mashen piglets (200×). One day, newborn; 28 days, weaning stage; and 70 days, end of nursey stage. Differences between two pig breeds were analyzed using Student’s *t*-test, *P < 0.05 and **P < 0.01 indicate significant difference. Differences among different time points per breed were identified using ANOVA, values with the same or no letter superscripts mean no significant difference (*P* > 0.05), while with different small letter superscripts mean significant difference at the level of 0.05, and with different capital letter superscripts mean significant difference at the level of 0.01.

To determine the physiological features of the ileum, we measured its villus length and crypt depth (Figure 1). We observed that the villus length of the ileum in the Jinfen White and Mashen piglets decreased from birth (1 day) to the end of nursery (70 days) stage, and there was a significant difference in the villus length between the two breeds on the 28 and 70 days (*P* < 0.05). Moreover, the villus length of the ileum in the Mashen piglets was higher than that in the Jinfen White piglets at all three stages, except at the end of the nursery stage (Figure 1A). In contrast, as depicted in Figure 1B, the crypt depth of the ileum in the Jinfen White and Mashen piglets at the end of the nursery stage were extremely significantly higher than in the other two stages (*P* < 0.01), and the crypt depth was significantly higher in the Mashen piglets than in the Jinfen White at the newborn (*P* < 0.01) and end of the nursery stages (*P* < 0.05). Furthermore, the villus/crypt ratio showed a downward trend, with no significant difference between the two breeds on the 1 and 70 days (Figure 1C).

Histological sections revealed that the villi of the ileum gradually became shorter with increasing age, but the transverse section of the villi became wider in the Jinfen White and Mashen piglets. However, intestinal folds were observed in the ileum of the Jinfen White piglets at birth, but not in the Mashen piglets. In addition, the ileum villi of the Jinfen White piglets were shorter than those of the Mashen piglets at birth, but in the Mashen piglets, the villi of the ileum were longer, and the surface area was larger than that in the Jinfen White piglets at the weaning stage and at the end of the nursery stage (Figure 1D).

### Change in Serum Levels of Antioxidant Factors at Different Developmental Stages

**FIGURE 2:**
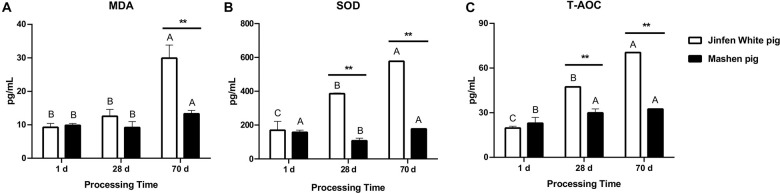
The change of antioxidant index in serum. **(A)** The content of MDA, **(B)** SOD, and **(C)** T-AOC in serum of Jinfen White and Mashen piglets. *P < 0.05, **P < 0.01, and ***P < 0.001 indicate significant difference between two pig breeds.

The small intestine is the main organ for food digestion and nutrient absorption and is composed of the mucous membrane and muscle layer. Some indicators in the blood are often associated with growth, immune responses, and gut health. To determine the association between serum biochemical indexes (especially antioxidant factors) and intestinal microbiota, we measured the content of MDA, SOD, and T-AOC using the blood samples of piglets at three developmental stages (*N* = 3). In general, the levels of MDA, SOD, and T-AOC in the serum of the Jinfen White and Mashen piglets increased over time, except for the MDA and SOD content of the Mashen piglets at the weaning stage (Figures 2A,B), of which the SOD and T-AOC increased by an extremely significant amount in the Jinfen White piglets (*P* < 0.01). Additionally, as described in Figure 2, the levels of the three indexes in the serum of the Jinfen White piglets were extremely significantly higher than those in the Mashen piglets at the end of nursing stage (*P* < 0.01). We also found that the blood levels of SOD and T-AOC in the Jinfen White piglets were extremely significantly higher than those in the Mashen piglets at the weaning stage and end of nursery stage (*P* < 0.01) (Figures 2B,C). These results demonstrated that with development, the oxidative stress response of the Jinfen White piglets may be more significant than that of the Mashen piglets, especially on the 70th day of the growth period, which indicated the end of nursery stage and start of the feeding.

### Ileum Microbiota Richness and Diversity of the Jinfen White and Mashen Piglets

**FIGURE 3:**
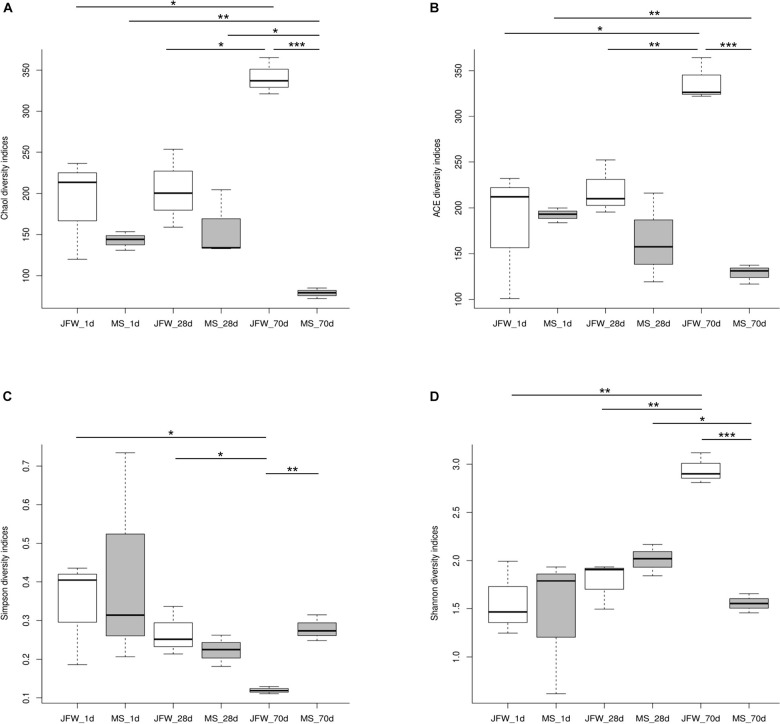
The ileum microbiota α diversity of Jinfen White and Mashen piglets. **(A)** Chao1 indices, **(B)** ACE indices, **(C)** Simpson indices, and **(D)** Shannon indices. *P < 0.05, **P < 0.01, and ***P < 0.001 indicate significant difference among different time points.

A total of 938,758 sequence reads were obtained from 18 ileum samples, with an average of 52,153 reads per sample (ranging from 32,195 to 71,847), covering 415,877,051 bp (Supplementary Table 3). After subsampling each sample to an equal sequencing depth (31,221 reads per sample) and clustering, 690 OTUs at 97% identity were obtained. From a taxonomic perspective, 21 phyla, 37 classes, 70 orders, 127 families, and 286 genera were identified in the ileum samples of 18 piglets. The alpha diversity indices are shown in Table 1 and Figure 3. Good’s coverage was at least 99% for all samples, suggesting that almost all bacteria in the ileum samples could be detected. For the Jinfen White piglets, the values of Chao1, ACE, and Shannon continuously increased and were significantly higher on the 70th day than on the 1st day and 28th day (*P* < 0.05), except for the Simpson indices (Figure 3D). However, the four indices did not change regularly in the Mashen piglets and did not reach the highest level at the endpoint (the 70th day). The changes in the four indices suggested that the richness and diversity increased during the Jinfen White piglet development, indicating that a subset of bacteria became dominant in the Jinfen White piglet’s ileum samples. In summary, the median values of the alpha diversity indices of the Mashen piglets were lower than those of the Jinfen White piglets (except on the 70th day of Simpson, and the 1st and 28th day of Shannon), revealing that the ileum of the Jinfen White piglets had a relatively richer microbial community diversity than the Mashen piglets.

**TABLE 1:** The alpha diversity indices of Jinfen White piglets and Mashen piglets.

**Alpha diversity indices**	**Sample ID**	**Developmental stages (day)**		
		**1**	**28**	**70**
Chao1	Jinfen White pig	189.95 ± 50.33	204.32 ± 38.68	341.18 ± 18.22
	Mashen pig	142.85 ± 9.25	157.15 ± 33.53	78.80 ± 5.26
Ace	Jinfen White pig	181.74 ± 57.77	219.27 ± 24.07	337.57 ± 18.97
	Mashen pig	192.27 ± 6.62	164.30 ± 39.90	128.45 ± 8.64
Shannon	Jinfen White pig	1.57 ± 0.31	1.77 ± 0.20	2.94 ± 0.13
	Mashen pig	1.44 ± 0.59	2.01 ± 0.13	1.55 ± 0.08
Simpson	Jinfen White pig	0.34 ± 0.11	0.27 ± 0.05	0.11 ± 0.007
	Mashen pig	0.42 ± 0.23	0.22 ± 0.03	0.28 ± 0.03
Coverage (%)	Jinfen White pig	99.94	99.93	99.9
	Mashen pig	99.89	99.94	99.96

**FIGURE 4:**
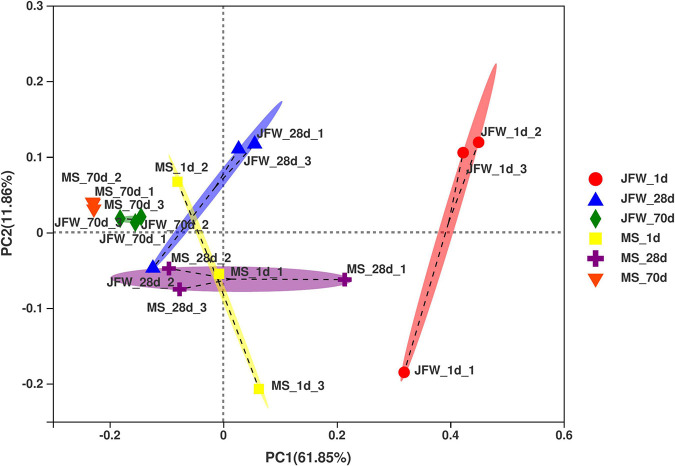
Principal coordinates analysis (PCoA) of the samples based on the OTU profiling.

Based on the relative abundance of the OUTs sequenced at the developmental stages of the two pig breeds, PCoA was used to analyze the differences in microbial community composition among the samples (Figure 4). As a result, the development of piglets was the first principal component, which explained 61.85% of the variation, indicating that development was the main factor affecting intestinal microbial changes. Interestingly, there were significant differences between the two breeds in the microbial variation trend. The microbial variation of the Jinfen White piglets was larger during the three developmental stages, while the microbial species of the Mashen piglets tended to be more stable during the entire development stage. Furthermore, microbial diversity was the second principal component, which explained 11.86% of the data variation, indicating that there were significant differences among individual piglets. The microorganisms of the Jinfen White piglets changed greatly before the weaning stage, and gradually stabilized at the nursery stage, while the microbes of the Mashen piglets were more similar from the newborn to the end of nursery stage. In summary, the intestinal microbiomes of different pig breeds were different, and the main factors affecting the stability of the intestinal microbiota of piglets were lactation and feed.

### Differences of the Ileum Microbial Taxa Represented in the Two Pig Breeds

**FIGURE 5:**
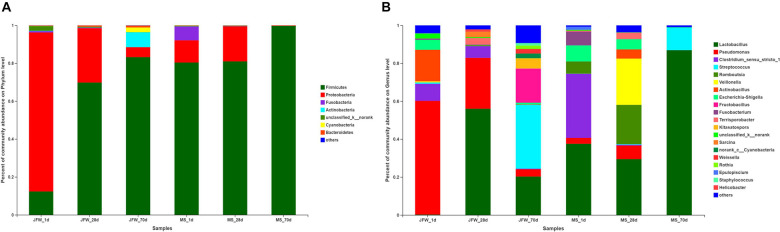
Characterization of ileum microbiota from Jinfen White and Mashen piglets. **(A)** Microbial community bar plot of phyla in piglet ileum. **(B)** Microbial community bar plot of genera in piglet ileum.

A total of 21 phyla were identified in the sequencing analysis, including Firmicutes, Proteobacteria, Actinobacteria, Fusobacteria, Cyanobacteria, and others (Figure 5A). Firmicutes and Proteobacteria were the major phyla during the development of the Jinfen White and Mashen piglets, accounting for ∼90% of all OTUs. As the growth time increased, the proportion of Firmicutes increased from 12.32 to 83.21%, and the percentage of Proteobacteria decreased from 83.93 to 5.26% in the ileum of the Jinfen White piglets. For the Mashen piglets, the ratio of Firmicutes showed no significant difference between the 1st day and 28th day, while it increased from 80.97 to 99.75% at the end of the nursing stage, and the proportion of Proteobacteria first increased and then decreased during the Mashen piglet’s development stages (Table 2).

**TABLE 2:** The ratio of identified phyla at different developmental stages of Jinfen White and Mashen piglets.

**Phylum**	**OUT_number**	**JFW_1d (%)**	**JFW_28d (%)**	**JFW_70d (%)**	**MS_1d (%)**	**MS_28d (%)**	**MS_70d (%)**
Firmicutes	252	12.32	69.82	83.21	80.36	80.97	99.75
Proteobacteria	106	83.93	28.66	5.26	11.75	18.57	0.23
Fusobacteria	5	0.69	0.31	0.04	7.28	0.02	0
Actinobacteria	54	0.20	0.04	7.83	0.05	0.12	0
Unclassified_k__norank	137	2.48	0.44	0.05	0.41	0.11	0
Cyanobacteria	9	0.11	0.04	2.48	0	0	0.01
Bacteroidetes	97	0.18	0.66	1	0.14	0.18	0.01
Others	30	0.1	0.03	0.12	0	0.03	0

Bacterial community dynamics were also evaluated based on changes in the relative abundance at the genus level during the growth of the Jinfen White and Mashen piglets (Figure 5B). A total of 286 bacterial genera were identified using HTS. At the developmental stage, *Pseudomonas* was the dominant genus in the ileum of Jinfen White piglets (59.97, 26.85, and 3.83%), and *Lactobacillus* was the major genus in the ileum of the Mashen piglets (37.54, 29.46, and 86.91%). At the weaning stage, *Lactobacillus* was the dominant genus in both the Jinfen White and Mashen piglets (56.03 and 29.46%, respectively) (Table 3). Moreover, *Streptococcus* and *Lactobacillus* were the dominant bacteria at the end of nursery period in the Jinfen White and Mashen piglets, respectively. These results indicate that the microbial composition changes dynamically during the growth and development period of piglets. In this study, except for the primary stage of the Jinfen White piglets, *Lactobacillus* was the core bacteria in the ileum of all samples, which is frequently observed in the guts of pigs (Valeriano et al., 2017; Gao et al., 2019a).

**TABLE 3:** The ratio of identified genera at different developmental stages of Jinfen White and Mashen piglets.

**Genus**	**OUT_number**	**JFW_1d (%)**	**JFW_28d (%)**	**JFW_70d (%)**	**MS_1d (%)**	**MS_28d (%)**	**MS_70d (%)**
*Lactobacillus*	22	0.23	56.03	20.27	37.54	29.46	86.91
*Pseudomonas*	4	59.97	26.85	3.83	3.13	7.17	0
*Clostridium_sensu_stricto_1*	5	9.00	6.17	0.32	33.80	0.44	0
*Streptococcus*	9	0.68	0.02	33.73	0.06	0.31	11.88
*Romboutsia*	1	0.05	0.48	0.06	6.42	20.70	0
*Veillonella*	3	0.55	0.02	0.03	0.01	24.39	0
*Actinobacillus*	5	16.65	0	0.14	0.01	4.88	0
*Escherichia–Shigella*	1	5.22	0.05	0.81	8.47	5.38	0.21
*Fructobacillus*	2	0	0	17.88	0	0	0
*Fusobacterium*	4	0.69	0.31	0.04	7.28	0.02	0
*Terrisporobacter*	1	0.03	3.39	0.20	0.50	3.43	0
*Kitasatospora*	1	0	0	5.30	0	0	0
*Unclassified_k__norank*	137	2.48	0.44	0.05	0.41	0.11	0
*Sarcina*	1	0	2.87	0	0.01	0	0
*Norank_c__Cyanobacteria*	3	0.05	0.02	2.47	0	0	0.01
*Weissella*	1	0	0	2.36	0	0	0
*Rothia*	2	0.10	0.01	1.83	0.04	0.02	0
*Epulopiscium*	1	0.05	0	0	1.58	0.01	0
*Staphylococcus*	3	0.05	0	1.31	0.01	0	0
*Helicobacter*	2	0	1.24	0	0	0	0
Others	482	4.17	2.11	9.35	0.75	3.67	0.97

### Predicted Encyclopedia of Genes and Genomes Pathways of Ileum Microbiota Between Jinfen White and Mashen Piglets

**FIGURE 6:**
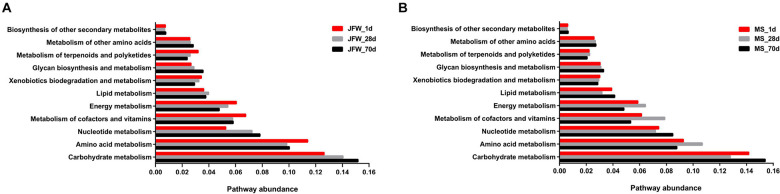
The abundance of metabolic pathways in microorganisms from ileum. **(A)** The pathway abundance of Jinfen White piglets and **(B)** Mashen piglets.

As the abundance of metabolic pathways in microorganisms determines the status of the ileum, microbial metabolism can provide further insight into the development of pigs. As shown in Figure 6, carbohydrate metabolism and amino acid metabolism were the main metabolic pathways in all samples of the two pig breeds. For the Jinfen White piglets (Figure 6A), the abundance of genera associated with amino acid metabolism began to decline after 28 days, and carbohydrate metabolism began to increase. For the Mashen piglets (Figure 6B), on day 28, although the carbohydrate metabolism began to decrease and the amino acid metabolism began to increase, the two types of metabolisms showed an opposite tendency after 70 days.

**FIGURE 7:**
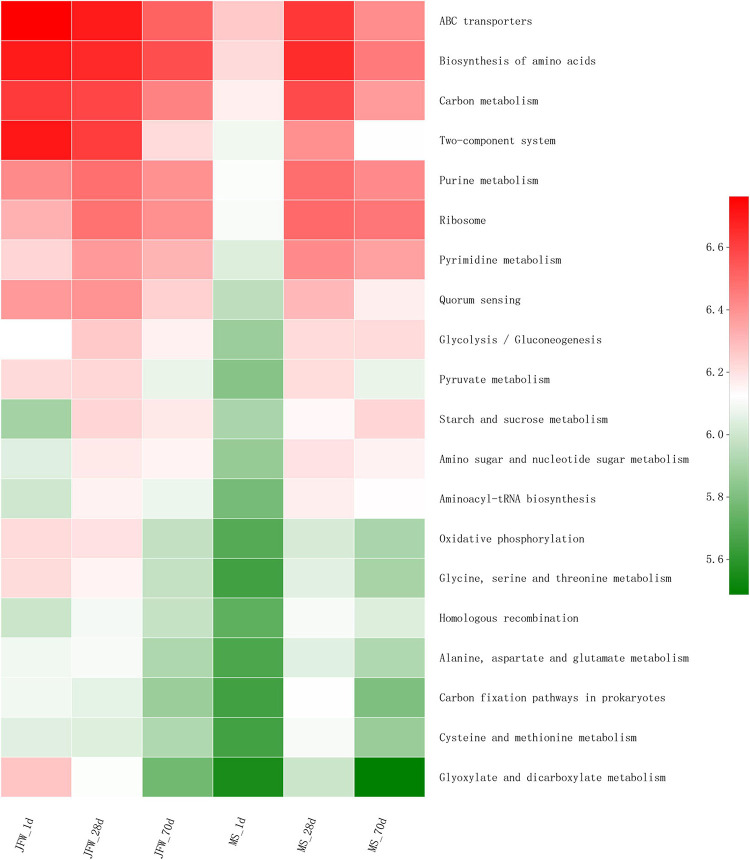
The top 20 KEGG pathway of ileum microbiota in Jinfen White piglets and Mashen piglets.

Several predicted KEGG pathways (top 20) were differentially identified in the ileum microbiota of the Jinfen White and Mashen piglets (Figure 7). All the top 20 KEGG pathways had the lowest abundance in the MS_1d group and had a relatively high abundance on the 28th day in the Jinfen White and Mashen piglets. The pathways in the JFW_28d and MS_28d groups were similar, and these pathways were also similar in the JFW_1d samples. For instance, the “Carbon metabolism” pathway was predicted at a lower level in the microbiota of the JFW_70d group than in that of the JFW_1d and JFW_28d, but at a higher level in the microbiota of the MS_70d group than in that of MS_1d.

### Correlation Between the Microbiota and Environmental Factors

**FIGURE 8:**
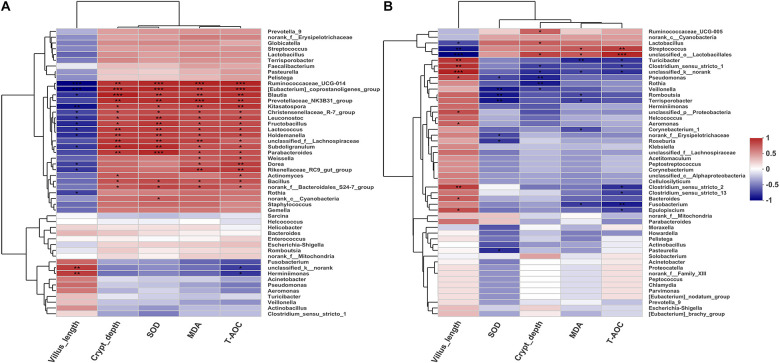
Spearman correlation coefficient between the ileum microbiota composition of the genera (top 50) and seven environmental factors of the Jinfen White and Mashen piglets. **(A)** Correlation between Jinfen White piglets and environmental variables and **(B)** correlation between Mashen piglets and environmental variables. Columns and rows represent environmental factors and genera, respectively. *R*-values are represented by colors, as indicated in the figure legend on the right side of the figure. *P < 0.05, **P < 0.01, and ***P < 0.001 indicate significant correlation between microbial genera and environmental variables.

To investigate the possible correlations between the microbial community structure and environmental factors, we analyzed the Spearman correlation coefficient between the ileum microbiota composition of the genera (top 50) and several physiological features (villus length, crypt depth, SOD, MAD, and T-AOC) of the Jinfen White and Mashen pigs by hierarchical clustering (Figure 8). For the Jinfen White piglets (Figure 8A), 15 genera, including *Ruminococcaceae_UCG-014*, *[Eubacterium]_coprostanoligenes_group*, *Blautia*, *Prevotellaceae_NK3B31_group*, *Kitasatospora*, *Christensenellaceae_R-7_group*, *Leuconostoc*, *Fructobacillus*, *Lactococcus*, *Holdemanella*, *unclassified_f__Lachnospiraceae*, *Subdoligranulum*, *Parabacteroides*, *Bacillus*, and *norank_f__Bacteroidales_S24-7_group* showed significantly positive correlations with the contents of SOD, MDA, and T-AOC in the serum as well as with the crypt depth (*P* < 0.05), whereas some of them (*Ruminococcaceae_UCG-014*, *[Eubacterium]_coprostanoligenes_group*, *Blautia*, *Kitasatospora*, *Christensenellaceae_R-7_group*, *Leuconostoc*, *Fructobacillus*, *Lactococcus*, *Holdemanella*, and *Subdoligranulum*) had remarkably negative correlations with villus length (*P* < 0.05). *Rothia* demonstrated a significant negative correlation with villus length (*P* < 0.05). However, *norank_c__Cyanobacteria* was only significantly positively correlated with SOD content in the serum of Jinfen White piglets. These results revealed positive correlation between the ileum microbiota at the genus level and serum biochemical index of Jinfen White piglets, except for *unclassified_k__norank* and *Herminiimonas*. Unlike the correlation in Jinfen White piglets, most of the bacteria were significantly negatively correlated with the serum biochemical indexes and crypt depth in the ileum of Mashen piglets except *Ruminococcaceae_UCG-005*, *Lactobacillus*, *Streptococcus*, and *unclassified_o__Lactobacillales*, which had significantly positive correlations with the contents of T-AOC, MDA, or crypt depth (*P* < 0.05) (Figure 8B). The serum content of T-AOC was significantly negatively correlated with *Clostridium_sensu_stricto_13* and *Clostridium_sensu_stricto_2* (*P* < 0.05). Furthermore, villus length was significantly positively correlated with *Turicibacter*, *Clostridium_sensu_stricto_1*, *unclassified_k__norank*, *Pseudomonas*, *unclassified_p__Proteobacteria*, *Aeromonas*, *Clostridium_sensu_stricto_2*, *Bacteroides*, and *Epulopiscium*. Interestingly, some bacteria had different correlations with environmental factors, while others showed comparable correlations in the two pig breeds. For instance, *Rothia* had a different correlation with all the environmental factors in the two pig breeds, while *Lactobacillus*, *Streptococcus*, *Fusobacterium*, and *Herminiimonas* were positively correlated with villus length and negatively correlated with other factors. These results suggest that the environmental and host factors (villus length, crypt depth, SOD, MAD, and T-AOC) may affect the formation of microbial community structures in the Jinfen White and Mashen piglets.

## Discussion

In recent years, several studies have greatly expanded our knowledge of the role of the intestinal microbiota in pigs by using next-generation sequencing technology (Han et al., 2017; Si et al., 2020). Thousands of microbes have been discovered that inhabit the gut of animals and form mutually beneficial relationships with their hosts thus playing an important role in maintaining health and resisting disease (Backhed et al., 2012; Hill and Round, 2021). Some studies have shown that during the development of piglets, the structure of the intestinal microflora and the α diversity are significantly changed, and the trend of this change is not consistent as it is affected by many factors such as time and heredity (Frese et al., 2015; Gao et al., 2019b). However, as a local hybrid breed of multiple pigs, the studies on the ileum microbiota of Jinfen White piglets have not yet been conducted. Thus, it is necessary to analyze the gut microbiota of the Jinfen White and Mashen pig breeds to identify the microbial and phenotypic differences and provide new insights. Early selection of breeding stock could improve the selection and achieve rapid genetic progress (Si et al., 2020). The goal of the present study was to investigate the features of ileum physiological structure and microbiota in three different growth stages of two pig breeds, including newborn piglets (1 day), weaning piglets (28 days), and the end of nursery piglets (70 days) of the Jinfen White and Mashen pig breeds, to elucidate the changes in the microbial structure and its correlation with phenotypic features.

In our previous study, the villus length and crypt depth of local pig (Shanxi Black pig) were higher than Large White pig (Gao et al., 2019b). Similarly, evaluation of the physiological features of the ileum in two pig breeds revealed that the villus length decreased with the development of piglets; however, in this study, the crypt depth increased and was significantly higher in the Mashen piglets than in the Jinfen White piglets at the newborn and the end of nursery stage. These studies suggest that to some extent the intestinal system of the local piglets (Shanxi Black and Mashen pig) is more developed than other breeds. For the Mashen piglets, the serum contents of MDA, SOD, and T-AOC were lower than those in the Jinfen White piglets. Accordingly, we inferred that the oxidative stress response of the Jinfen White piglets may be more significant than that of the Mashen piglets during their development. We then focused on the composition and function of microbial communities in the ileum of these two pig breeds at three developmental stages (newborn, weaning, and end of nursery).

Previous studies have indicated significant differences in fecal and intestinal microbial composition among pigs at different developmental stages (Ke et al., 2019; Kumar et al., 2019; Shao et al., 2021). With the development in pigs, the intestinal microbiota structure changes significantly, as well as the α diversity, but the trend is not consistent, as it is affected by several factors including age and genetics (Frese et al., 2015). Our study also found that the ileum microbiota of the two pig breeds was similar, but exhibited significant differences at the three points. This indicates that the formation and development of gut microbiota is influenced by the feed, environmental factors, and developmental stage, with developmental stage being the important factor causing the differences in gut microbiota composition. The diversity of ileum microbiota was less in Mashen piglets than that of the Jinfen White piglets, but was more stable, suggesting that this local pig breed has a strong ability to adapt to environmental changes.

Firmicutes, Bacteroidetes and Proteobacteria are the main bacteria inhabiting the piglet gut (Mach et al., 2015). Firmicutes can help to extract energy from ingested food (Komaroff, 2017), and the abundance of Firmicutes in the ileum of Mashen piglets (especially newborns) was significantly higher than that of the Jinfen White piglets, suggesting that the high abundance of Firmicutes may promote the fat accumulation in Mashen piglets. Studies have also found that the *Clostridium*, *Pseudomonas*, and *Lactobacillus* are the main genera in the intestine (Zoetendal et al., 2012; Chen et al., 2017). *Lactobacillus* plays an important role in the metabolism of plant food (Filannino et al., 2018), and could impart anti-cancer and anti-inflammatory effects (Fernández et al., 2016). In this study, the abundance of *Lactobacillus* in the ileum of Mashen piglets was relatively high, especially at the end of nursery stage, and thus can play an important role in maintaining intestinal health and nutrient digestion and absorption. We found that the gut microbiota composition was associated with specific physiological features (including the villus length and crypt depth) and biochemical indicators of serum. Interestingly, the villus length negatively correlated with most microbiota in Jinfen White piglets, whereas positively correlated with ileum microorganisms in the Mashen piglets. In particular, *Actinobacillus* positively correlated with crypt depth and negatively correlated with villus length in the Jinfen White piglets, which is different from Large White and Shanxi Black pigs. Therefore, this study revealed that different bacterial genera have a distinguishing correlation with the host and environment in both breeds of piglets.

The Mashen pig is a local breed in China, while the Jinfen White pig is a hybrid of a Chinese local breed and foreign breeds. This study should include the Large White pig or Landrace pig to better clarify the influence of differences in genetic composition on intestinal microbiota. However, since these breeds are not raised in the same farm, only Mashen pigs and Jinfen White pigs were chosen for the comparative analysis at the beginning of this study. Thus, the influence of genetic composition of different breeds on the intestinal microbial diversity will be studied in the future.

In conclusion, this study showed differences in the ileum microbiomes during the three developmental stages of Jinfen White and Mashen piglets. The physical features and serum of the Jinfen White and Mashen piglets were different. With increasing age, the gut microbial diversity increased and the proportion of Firmicutes increased, whereas the proportion of Proteobacteria decreased in both the pig breeds. Therefore, as a cultivated breed, the oxidative stress response and microbiota diversity of Jinfen White piglets may be more significant than those of Mashen piglets. This study compared the structural and functional changes of the microbes of two breeds of piglets and provided an important reference for future research on nutritional regulation by the microbiota.

## Data Availability Statement

The datasets presented in this study can be found in online repositories. The names of the repository/repositories and accession number(s) can be found below: https://www.ncbi.nlm.nih.gov/, SRP321842.

## Ethics Statement

The animal study was reviewed and approved by the Animal Care and Use Committee of Shanxi Agricultural University.

## Author Contributions

PG, XG, GC, and BL designed this study. YL, YM, SW, CC, and YZ took the ileum of Mashen and Jinfen White piglets. YL and SW prepared the DNA samples. CL, GL, and YY performed the data analysis. CL, PG, XG, and SK wrote the manuscript. All authors contributed to the article and approved the final version.

## Conflict of Interest

The authors declare that the research was conducted in the absence of any commercial or financial relationships that could be construed as a potential conflict of interest.

## Publisher’s Note

All claims expressed in this article are solely those of the authors and do not necessarily represent those of their affiliated organizations, or those of the publisher, the editors and the reviewers. Any product that may be evaluated in this article, or claim that may be made by its manufacturer, is not guaranteed or endorsed by the publisher.

## Footnotes

1
http://www.drive5.com/usearch/

2
http://www.arb-silva.de

3
https://www.mothur.org

## References

[B1] Asshauer K. P. Wemheuer B. Daniel R. Meinicke P. (2015). Tax4Fun: predicting functional profiles from metagenomic 16S rRNA data. *Bioinformatics* 31 2882–2884. 10.1093/bioinformatics/btv287 25957349 PMC4547618

[B2] Backhed F. Fraser C. M. Ringel Y. Sanders M. E. Sartor R. B. Sherman P. M. (2012). Defining a healthy human gut microbiome: current concepts, future directions, and clinical applications. *Cell Host Microbe* 12 611–622. 10.1016/j.chom.2012.10.012 23159051

[B3] Caporaso J. G. Kuczynski J. Stombaugh J. Bittinger K. Bushman F. D. Costello E. K. (2010). QIIME allows analysis of high-throughput community sequencing data. *Nat. Methods* 7 335–336. 10.1038/nmeth.f.303 20383131 PMC3156573

[B4] Chen H. Mao X. B. Che L. Q. Yu B. He J. Yu J. (2014). Impact of fiber types on gut microbiota, gut environment and gut function in fattening pigs. *Anim. Feed Sci. Technol.* 195 101–111. 10.1016/j.anifeedsci.2014.06.002

[B5] Chen L. M. Xu Y. S. Chen X. Y. Fang C. Zhao L. P. Chen F. (2017). The maturing development of gut microbiota in commercial piglets during the weaning transition. *Front. Microbiol.* 8:1688. 10.3389/fmicb.2017.01688 28928724 PMC5591375

[B6] Cui W. T. Xiao G. J. Jiang S. W. Qian L. L. Cai C. B. Li B. (2019). Effect of ZFN-edited myostatin loss-of-function mutation on gut microbiota in Meishan pigs. *PLoS One* 14:e0210619. 10.1371/journal.pone.0210619 30645618 PMC6333347

[B7] Fernández J. Redondo-Blanco S. Gutiérrez-del-Río I. Miguélez E. M. Villar C. J. Lombó F. (2016). Colon microbiota fermentation of dietary prebiotics towards short-chain fatty acids and their roles as anti-inflammatory and antitumour agents: a review. *J. Funct. Foods* 25 511–522. 10.1016/j.jff.2016.06.032

[B8] Filannino P. Di Cagno R. Gobbetti M. (2018). Metabolic and functional paths of lactic acid bacteria in plant foods: get out of the labyrinth. *Curr. Opin. Biotechnol.* 49 64–72. 10.1016/j.copbio.2017.07.016 28830019

[B9] Frese S. A. Parker K. Calvert C. C. Mills D. A. (2015). Diet shapes the gut microbiome of pigs during nursing and weaning. *Microbiome* 3:28. 10.1186/s40168-015-0091-8 26167280 PMC4499176

[B10] Gao P. F. Liu Y. D. Le B. Y. Qin B. Y. Liu M. Zhao Y. Y. (2019b). A comparison of dynamic distributions of intestinal microbiota between Large White and Chinese Shanxi Black pigs. *Arch. Microbiol.* 201 357–367. 10.1007/s00203-019-01620-4 30673796

[B11] Gao P. F. Guo Y. L. Zhang N. F. Zhang W. F. Wang H. J. Guo X. H. (2019a). Characterization and comparisons of microbiota in different intestinal segments between adult Chinese Shanxi Black Pigs and Large White Pigs. *Ann. Microbiol.* 69 447–456. 10.1007/s13213-018-1430-3

[B12] Gill S. K. Rossi M. Bajka B. Whelan K. (2021). Dietary fibre in gastrointestinal health and disease. *Nat. Rev. Gastroenterol. Hepatol.* 18 101–116. 10.1038/s41575-020-00375-4 33208922

[B13] Guevarra R. B. Hong S. H. Cho J. H. Kim B. R. Shin J. Lee J. H. (2018). The dynamics of the piglet gut microbiome during the weaning transition in association with health and nutrition. *J. Anim. Sci. Biotechnol.* 9:54. 10.1186/s40104-018-0269-6 30069307 PMC6065057

[B14] Guevarra R. B. Lee J. H. Lee S. H. Seok M. J. Kim D. W. Kang B. N. (2019). Piglet gut microbial shifts early in life: causes and effects. *J. Anim. Sci. Biotechnol.* 10:1. 10.1186/s40104-018-0308-3 30651985 PMC6330741

[B15] Han G. G. Lee J. Y. Jin G. D. Park J. Choi Y. H. Chae B. J. (2017). Evaluating the association between body weight and the intestinal microbiota of weaned piglets via 16S rRNA sequencing. *Appl. Microbiol. Biotechnol.* 101 5903–5911. 10.1007/s00253-017-8304-7 28523395

[B16] Hill J. H. Round J. L. (2021). SnapShot: microbiota effects on host physiology. *Cell* 184 2796–2796.e1. 10.1016/j.cell.2021.04.026 33989551

[B17] Hillman E. T. Lu H. Yao T. Nakatsu C. H. (2017). Microbial ecology along the gastrointestinal tract. *Microbes Environ.* 32 300–313. 10.1264/jsme2.ME17017 29129876 PMC5745014

[B18] Holman D. B. Brunelle B. W. Trachsel J. Allen H. K. (2017). Meta-analysis to define a core microbiota in the swine gut. *mSystems* 2:e00004–e17. 10.1128/mSystems.00004-17 28567446 PMC5443231

[B19] Judkins T. C. Archer D. L. Kramer D. C. Solch R. J. (2020). Probiotics, nutrition, and the small intestine. *Curr. Gastroenterol. Rep.* 22:2. 10.1007/s11894-019-0740-3 31930437

[B20] Ke S. L. Fang S. M. He M. Z. Huang X. C. Yang H. Yang B. (2019). Age-based dynamic changes of phylogenetic composition and interaction networks of health pig gut microbiome feeding in a uniformed condition. *BMC Vet. Res.* 15:172. 10.1186/s12917-019-1918-5 31126262 PMC6534858

[B21] Komaroff A. L. (2017). The microbiome and risk for obesity and diabetes. *JAMA* 317 355–356. 10.1001/jama.2016.20099 28006047

[B22] Kumar H. Park W. Srikanth K. Choi B. H. Cho E. S. Lee K. T. (2019). Comparison of bacterial populations in the ceca of swine at two different stages and their functional annotations. *Genes (Basel)* 10:382. 10.3390/genes10050382 31137556 PMC6562920

[B23] Li M. Zhang N. Zhang W. F. Hei W. Cai C. B. Yang Y. (2021). Comprehensive analysis of differentially expressed circRNAs and ceRNA regulatory network in porcine skeletal muscle. *BMC Genomics* 22:320. 10.1186/s12864-021-07645-8 33932987 PMC8088698

[B24] Liu Y. Zheng Z. J. Yu L. H. Wu S. Sun L. Wu S. L. (2019). Examination of the temporal and spatial dynamics of the gut microbiome in newborn piglets reveals distinct microbial communities in six intestinal segments. *Sci. Rep.* 9:3453. 10.1038/s41598-019-40235-z 30837612 PMC6400902

[B25] Mach N. Berri M. Estelle J. Levenez F. Lemonnier G. Denis C. (2015). Early-life establishment of the swine gut microbiome and impact on host phenotypes. *Environ. Microbiol. Rep.* 7 554–569. 10.1111/1758-2229.12285 25727666

[B26] Magoc T. Salzberg S. L. (2011). FLASH: fast length adjustment of short reads to improve genome assemblies. *Bioinformatics* 27 2957–2963. 10.1093/bioinformatics/btr507 21903629 PMC3198573

[B27] McCormack U. M. Curiao T. Buzoianu S. G. Prieto M. L. Ryan T. Varley P. (2017). Exploring a possible link between the intestinal microbiota and feed efficiency in pigs. *Appl. Environ. Microbiol.* 83:e00380-17. 10.1128/AEM.00380-17 28526795 PMC5514681

[B28] Shao M. Y. Wang Z. X. He Y. Z. Tan Z. Zhang J. B. (2021). Fecal microbial composition and functional diversity of Wuzhishan pigs at different growth stages. *AMB Express* 11:88. 10.1186/s13568-021-01249-x 34117938 PMC8197691

[B29] Si J. L. Feng L. L. Gao J. Y. Huang Y. Zhang G. J. Mo J. Y. (2020). Evaluating the association between feed efficiency and the fecal microbiota of early-life Duroc pigs using 16S rRNA sequencing. *AMB Express* 10:115. 10.1186/s13568-020-01050-2 32562009 PMC7305293

[B30] Valeriano V. D. Balolong M. P. Kang D. K. (2017). Probiotic roles of Lactobacillus sp. in swine: insights from gut microbiota. *J. Appl. Microbiol.* 122 554–567. 10.1111/jam.13364 27914202

[B31] Wang X. F. Tsai T. C. Deng F. L. Wei X. Y. Chai J. M. Knapp J. (2019). Longitudinal investigation of the swine gut microbiome from birth to market reveals stage and growth performance associated bacteria. *Microbiome* 7:109. 10.1186/s40168-019-0721-7 31362781 PMC6664762

[B32] Yang L. N. Bian G. R. Su Y. Zhu W. Y. (2014). Comparison of faecal microbial community of lantang, bama, erhualian, meishan, xiaomeishan, duroc, landrace, and yorkshire sows. *Asian-Australas. J. Anim. Sci.* 27 898–906. 10.5713/ajas.2013.13621 25050029 PMC4093183

[B33] Yang Y. Liu Y. D. Liu J. Wang H. Z. Guo Y. L. Du M. (2021). Composition of the fecal microbiota of piglets at various growth stages. *Front. Vet. Sci.* 8:661671. 10.3389/fvets.2021.661671 34336969 PMC8319241

[B34] Zhao W. J. Wang Y. P. Liu S. Y. Huang J. J. Zhai Z. X. He C. (2015). The dynamic distribution of porcine microbiota across different ages and gastrointestinal tract segments. *PLoS One* 10:e0117441. 10.1371/journal.pone.0117441 25688558 PMC4331431

[B35] Zoetendal E. G. Raes J. van den Bogert B. Arumugam M. Booijink C. C. Troost F. J. (2012). The human small intestinal microbiota is driven by rapid uptake and conversion of simple carbohydrates. *ISME J.* 6 1415–1426. 10.1038/ismej.2011.212 22258098 PMC3379644

